# Prognostic Radiological Tools for Clinical Stage IA Pure Solid Lung Cancer

**DOI:** 10.3390/curroncol28050328

**Published:** 2021-09-30

**Authors:** Takeo Nakada, Yusuke Takahashi, Noriaki Sakakura, Hiroshi Iwata, Takashi Ohtsuka, Hiroaki Kuroda

**Affiliations:** 1 Department of Thoracic Surgery, Aichi Cancer Center Hospital, Aichi 464-8681, Japan; y.takahashi@aichi-cc.jp (Y.T.); nsakakura@aichi-cc.jp (N.S.); h-kuroda@aichi-cc.jp (H.K.); 2 Department of Surgery, Division of Thoracic Surgery, The Jikei University School of Medicine, Tokyo 105-8471, Japan; t-oh@remus.dti.ne.jp; 3 East Nagoya Radiological Diagnosis Foundation, Aichi 464-0044, Japan; h-iwata@nagoya-pet.or.jp

**Keywords:** lung cancer, pure solid tumor, positron emission tomography, computed tomography, prognosis

## Abstract

In this study, we analyzed prognostic radiological tools and surgical outcomes for radiologically pure solid adenocarcinomas (AD) and squamous cell carcinoma (SQ) in clinical stage IA. We retrospectively investigated 130 patients who underwent surgical resections. We assessed the predictive risk factors for recurrence and pathological lymph node metastasis (LNM). There was no statistical difference in recurrence free survival (RFS) or cancer-specific survival (CSS) between AD and SQ groups (*p* = 0.642 and *p* = 0.403, respectively). In the whole cohort, tumor size on lung window and mediastinal settings, and tumor disappearance ratio using high-resolution computed tomography (HRCT) were not prognostic parameters (*p* = 0.127, 0.066, and 0.082, respectively). The maximal standardized uptake value (SUVmax) using positron emission tomography-CT was associated with recurrence (*p* = 0.016). According to the receiver operating characteristic curve, the cut-off value of SUVmax for recurrence was 4.6 (*p* = 0.016). The quantitative continuous variables using any radiological tools were not associated with LNM. However, tumor diameter on mediastinal setting ≥8 mm with SUVmax ≥2.4 could be a risk factor for LNM. Pure solid AD and SQ were equivalent for the RFS and CSS. SUVmax was useful to predict recurrence. The tumor diameter on a mediastinal setting and SUVmax were useful in predicting pathological LNM.

## 1. Introduction

Predicting oncological behaviors is important for surgical planning, aggressive surveillance, aggressive adjuvant, or neoadjuvant therapy. Since the adoption of the tumor–node–metastasis (TMN) cancer staging version eight, numerous studies predicting the prognostic capability of radiological tools using high-resolution computed tomography (HRCT) for stage I non-small cell lung cancer (NSCLC) have been published. Several authors considered that the presence of ground-grass opacity (GGO) had independently good radiological signatures for prognosis using HRCT in both clinical and pathological T1N0-Staged NSCLC [1,2,3,4,5]. However, the prognostic radiological tools regarding pure solid nodules in early stages are still unknown. In primary lung cancers, whereas most part solid nodules are adenocarcinomas (AD), pure solid nodules are mainly composed of AD and squamous cell carcinoma (SQ). Previously, some authors assessed the prognosis between AD and SQ. In stage I, SCC showed faster response atter stereotactic body radiotherapy than AD, although three- and four-year local control rates were similar [6]. Survival outcomes after segmentectomy or wedge resection were generally comparable for stage I ≤3 cm invasive AD and SQ [7]. However, in the pure solid type, their biological behavior was unclear. In this study, we evaluated prognostic radiological tools and surgical outcomes for radiologically pure solid stage IA, AD, and SQ. Subsequently, we evaluated the predictive risk factors for the pathological lymph node metastasis (LNM) in patients who underwent lobectomy with mediastinal lymph node dissection.

## 2. Materials and Methods

### 2.1. Patients

This study retrospectively reviewed the data patients who underwent pulmonary resection for clinical stage IA pure solid AD and SQ diagnosed at our institution between January 2012 and December 2016. GGOs were defined as areas with a slight increase in density on CT images that did not obscure the bronchi and blood vessels in the lung. A pure solid tumor was defined as a tumor without GGO on lung window view using HRCT. In this study, all patients were examined using positron emission tomography-CT (PET-CT) two months before surgery in the Higashi Nagoya imaging center. This study was performed in line with the principles of the Declaration of Helsinki. The data were prospectively collected, registered in a database, and approved by the Review Board of Aichi Cancer Center (approval number: 2020-1-704).

### 2.2. Data

We collected data on the following patient characteristics: age, sex, smoking history, body mass index, and spirometry test results (including percent of vital capacity and forced expiratory volume in 1 s as a percent of forced vital capacity). CEA and CYFRA were evaluated as tumor makers. Whole tumor size on lung window setting (WTS), diameter on mediastinal window setting (MD), and tumor disappearance ratio (TDR) were the parameters observed on HRCT. The lung window was set at a window level of −500 Hounsfield unit (HU) and a width of 1500 HU. The mediastinal window was set at a window level of 30 and a width of 330 HU. TDR (%) was calculated as follows: 100 × (1 − (solid portion size in the mediastinal window/total tumor size in the lung window)). PET-CT was performed in all patients based on stable glycemic control. The maximal standardized uptake value (SUVmax) is a representative radiological tool used to measure tumor aggressiveness in typical clinical practice. The selected surgical procedure for each patient (lobectomy, sublobar resection, or mediastinal lymph node dissection) was also included in the operative variables. Mediastinal lymph node dissection was performed based on selective mediastinal lymph node dissection [8]. Lymph node dissection or sampling was not performed in pulmonary wedge resections. Pathological variables included pathological tumor size, lymphovascular invasion, pleural invasion (pl2), histological low grade, and lymph node metastasis. Adjuvant chemotherapy experience was included as well. The final diagnoses were determined based on a postoperative pathological evaluation. The histological assessment was performed according to the World Health Organization histological classification. The TNM staging was introduced according to the eighth edition of the Union for International Cancer Control Classification [9,10]. The surgical outcomes included cancer recurrence rate, recurrence free survival (RFS), overall survival (OS), and cancer-specific survival (CSS). We defined the survival interval as the time between the date of surgery and the date of final follow-up, cancer recurrence, or death. Follow-up was recorded as per standard of care. The routine screening examinations using tumor markers and chest and abdominal CT were performed every four to six months for the first five years and annually every year thereafter. Cerebellar magnetic resonance imaging, bone scintigraphy, and PET-CT were performed on demand.

The exclusion criteria were as follows: lung cancer with GGO upon HRCT, patients treated for previous lung cancer, incomplete resection, histological types excluding AD or SQ, neoadjuvant chemotherapy, and incomplete data. According to the American College of Chest Physicians guidelines, as well as the French guidelines, PET is not indicated for solitary pulmonary nodules less than 8–10 mm in diameter since the threshold indicates the significant risk of false-negative findings [11,12,13]. Therefore, we also excluded tumors of 10 mm or less. The involvement of the mediastinal lymph node was assessed using CT enlargement (short diameter >10 mm), focally increased FDG uptake compared with normal background activity, or SUVmax >2.5 on FDG-PET, which was diagnosed by radiological specialists at the Higashi Nagoya imaging center.

### 2.3. Statistical Analysis

The statistical analysis was performed using SPSS version 21.0 software (IBM Corp., Armonk, NY, USA). A two-sided *p*-value < 0.05 was considered statistically significant. We first compared multivariable parameters and prognosis between the AD and SQ groups. Then, we assessed the prognostic risk factors in relation to the multivariable parameters between the recurrence and non-recurrence groups. On subgroup analysis, we evaluated predictive risk factors for pathological LNM and non-LNM in patients who underwent lobectomy with mediastinal lymph node dissection.

The quantitative values were expressed as the median or average ± standard deviation with interquartile range. We compared their significant differences between groups using Student *t*-test and the Mann–Whitney U test. The categorical variables were compared using the χ^2^ and Fisher’s exact tests. The RFS, OS, and CSS were estimated using the Kaplan–Meier method, and the differences between groups were assessed using the log-rank test.

## 3. Results

### 3.1. Patient Characteristics

**Figure 1:**
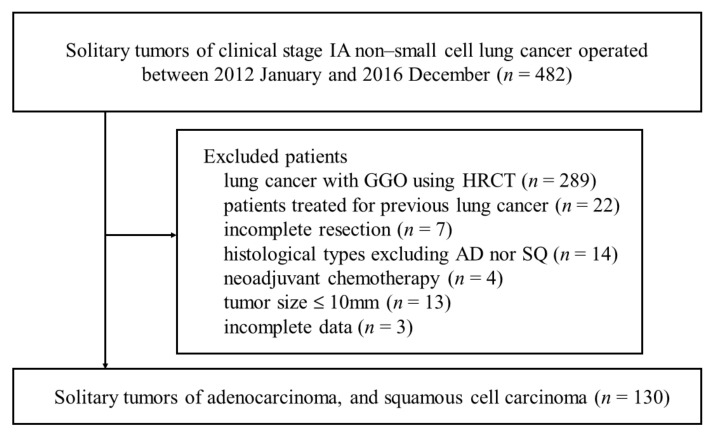
Patient’s flow chart.

During the study period, 482 patients underwent pulmonary resection for clinical stage IA, AD, or SQ that was clinically diagnosed using PET-CT. One hundred and thirty patients met the criteria and were included in the study. Excluded patients are shown in Figure 1. Among other histological types, five patients had typical carcinoid, one had pleomorphic carcinoma, two had adenosquamous cell carcinoma, three had large cell carcinoma, and three had large cell neuroendocrine carcinoma. According to the medical records, there were no patients with EBUS before surgery in this cohort. The median follow-up period was 50.7 ± 19.3 months (range, 2–100 months). The patients’ median age was 69 years; there were 69 men and 61 women. Pathologically, AD was diagnosed in 96 (73.8%) patients, and SQ in 34 (26.2%). Nineteen patients had cancer recurrence (14.6%).

### 3.2. Analysis between the AD and SQ Groups

Table 1 presents the compared multivariable parameters and prognosis between the AD and SQ groups. On univariate analysis, elderly age, higher incidence in the male sex, significant smoking history, lower TDR, and higher value of SUVmax were statistically associated with the SQ group (all *p* < 0.05). Other parameters including pathological findings were not different between both groups. Sakakura suggested that MD ≤ 2 mm using HRCT predicted minimally invasive adenocarcinoma with high specificity (94.5%) in AD [14]. Our study involved two nodules of AD with MD ≤ 2 mm, which were diagnosed as pathologically invasive AD.

**Figure 2:**
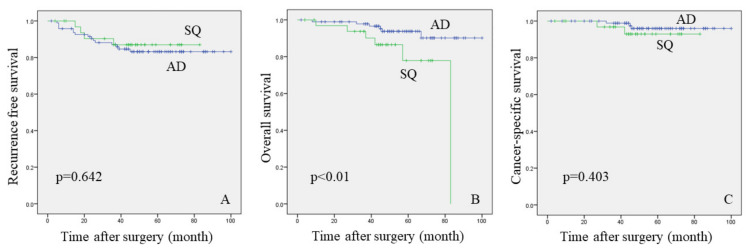
Kaplan-Meier curves ((**A**) recurrence free survival, (**B**) overall survival, and (**C**) cancer-specific survival). Differences in survivor function between patient groups were assessed using the log-rank test. AD, adenocarcinoma; SQ, squamous cell carcinoma.

Cancer recurrence rates were 15.8% and 11.8% in the AD and SQ groups, respectively (*p* = 0.408). In the AD group, the three- and five-year recurrence-free rates were 87% and 83.2%, respectively. On the other hand, the recurrence free rates in the SQ group were 87% for both three- and five-year time periods. There was no statistical deference in cancer recurrence between both groups (Figure 2A, *p* = 0.642, log-rank test). In the AD group, the three- and five-year overall survival rates were 97.8% and 83.2%, respectively. In the SQ group, the three- and five-year overall survival rates were 93.8% and 77.9%, respectively. Shorter OS was associated with SQ group (Figure 2B, *p* < 0.01, log-rank test). However, for CSS, the three- and five-year cancer-specific survival rates were 98.9 and 96%, respectively in the AD group; the three- and five-year cancer-specific survival rates were 96.8 and 92.9%, respectively, in the SQ group (Figure 2C, *p* = 0.403, log-rank test).

### 3.3. Analysis for the Recurrence

The statistical findings regarding recurrence and non-recurrence groups are shown in Table 2. Nineteen patients had cancer recurrence (14.6%). There were 14 patients with intrathoracic recurrence (73.7%), while distant metastasis (26.3%) was seen in the remaining five patients. Intrathoracic recurrence included intrathoracic lymph node metastasis (nine patients), status post wedge resection (two patients) or lobectomy (seven patients), intrapulmonary metastasis (three patients), and intrathoracic dissemination (two patients).

The patient’s characteristics, spirometry test results, tumor markers, and surgical procedures were not different between both groups. In HRCT findings, WTS, MD, and TDR were not associated with recurrence (*p* = 0.127, 0.066, and 0.082, respectively). A higher value of SUVmax was a risk factor of cancer recurrence (*p* = 0.016). Pathological type was not associated with cancer recurrence, but lymph node metastasis was a risk factor for cancer recurrence (*p* = 0.007). Cancer recurrence was also associated with shorter CSS (log-rank test: *p* < 0.01).

### 3.4. The Cut-Off Value of Suvmax Associated with Cancer Recurrence

**Figure 3:**
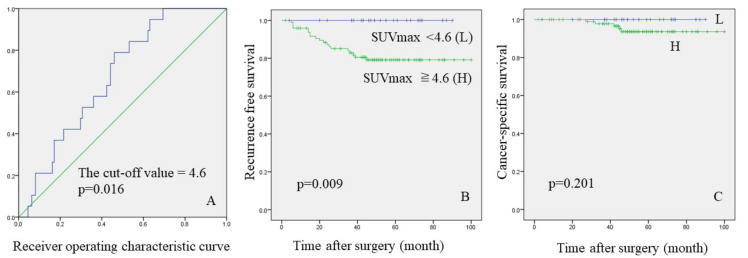
(**A**): the receiver operating characteristic curve for predicting cancer recurrence. Kaplan-Meier curves ((**B**): recurrence free survival, (**C**): cancer-specific survival).

In this study, SUVmax was the most useful predictive radiological tool for cancer recurrence. According to the area under the receiver operating characteristic curve, the optimal cut-off value for predicting cancer recurrence was 4.6 (area under the curve = 0.674; sensitivity = 94.7%; specificity = 69.4%; 95% confidence interval = 0.564–0.780; *p* = 0.016) (Figure 3A). While the three- and five-year recurrence free rates were both 100% in the SUVmax <4.6 group, the three- and five-year recurrence free rates were 80.5 and 79.1%, respectively, in the SUVmax ≥4.6 group (Figure 3B, log-rank test: *p* = 0.009). However, the value of SUVmax was not useful to predict CSS (Figure 3C, log-rank test: *p* = 0.201). 

**Figure 4:**
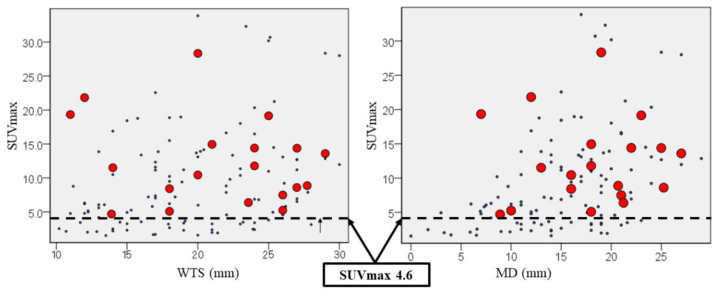
SUVmax ≥4.6 was useful to predict the recurrence regardless of HRCT parameters. Red spots revealed cancer recurrence. WTS; whole tumor size on lung window setting, and MD; diameter on mediastinal window setting.

The scatter diagrams of the recurrence for radiological tools are revealed in Figure 4, which showed that SUVmax ≥4.6 was useful to predict the recurrence regardless of WTS and MD.

### 3.5. Analysis for the Lymph Node Metastasis

**Figure 5:**
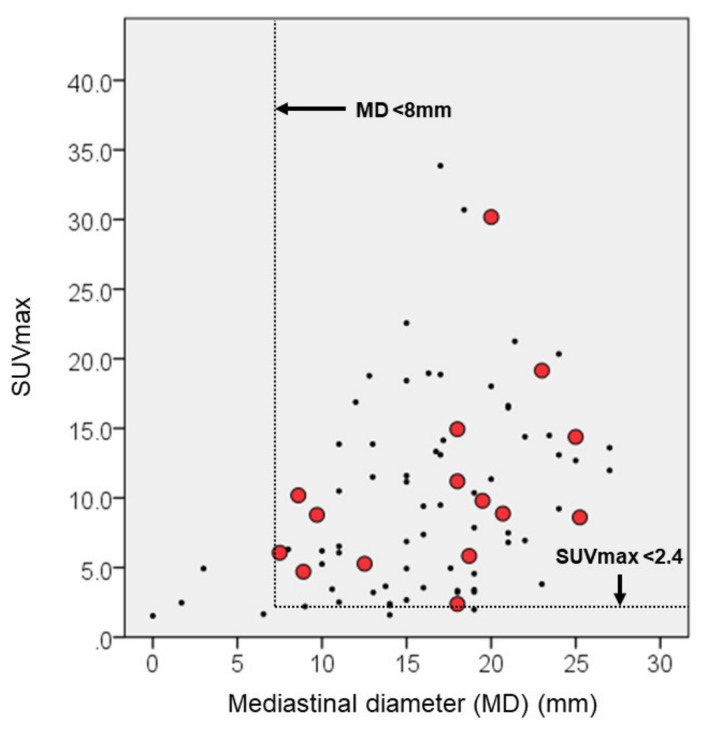
Diameter on mediastinal window setting (MD) ≥8 mm with SUVmax ≥2.4 could be a risk factor for lymph node metastasis. Red spots revealed cancer recurrence.

We evaluated risk factors for pathological LNM and non-LNM in 83 patients who underwent lobectomy with mediastinal lymph node dissection. Table 3 demonstrates comparable multivariable parameters between the LNM and non-LNM groups. There was no statistical deference in total number of excised lymph nodes between both groups (*p* = 0.803). A high value of CYFRA and lymphovascular invasion were the only statistical risk factors for LNM (*p* = 0041, and 0.017, respectively). The quantitative continuous variables using any radiological tools were not associated with LNM. However, the tumor diameter on the mediastinal setting ≥8 mm with SUVmax ≥2.4 could be risk factors for LNM (Figure 5).

## 4. Discussion

In primary lung cancers, pure solid nodules are composed of multiple pathological variants, whereas most part solid nodules are adenocarcinomas (AD). In this study, 90.3% of pure solid lung nodules on high-resolution computed tomography (HRCT) consisted of adenocarcinoma and squamous cell carcinoma (SQ). Their biological behavior was unclear. Therefore, we focused on investigating the prognostic role of radiological tools for clinical stage IA pure solid AD and SQ.

First, we evaluated the clinicopathological differences and prognosis between these two histological types. In radiological findings, lower tumor disappearance ratio (TDR) and higher maximal value of standardized uptake (SUVmax) were statistically associated with the SQ group in comparison with the AD group (all *p* < 0.05). We considered that the various pathologic subtypes for AD may reflect on the higher TDR. In positron emission tomography-CT (PET-CT), higher value of SUVmax was related with the SQ group. In this study, AD and SQ had statistically similar prognosis. We considered that other effects, apart from the metabolic activity, caused the difference in the SUVmax. Previous reports revealed that the higher expression of glucose transporter type 1 impacted the difference of SUV in SQ than AD [15,16,17]. Sakakura suggested that the diameter on the mediastinal window setting using HRCT (MD) ≤2 mm predicted minimally invasive adenocarcinoma (MIA) with high specificity (94.5%) to evaluate 360 completely resected AD [14]. Our cohort included two nodules of AD with MD ≤2 mm, which were diagnosed as pathologically invasive AD. For radiological pure solid tumors, small MD size should not be indicated as predictive of MIA.

There was no difference in recurrence free survival (RFS) and cancer-specific survival (CSS) between AD and SQ groups (log-rank test: *p* = 0.642, and 0.403). Ito et al. evaluated 150 patients with only solid type AD and SQ <3 cm in diameter on HRCT who had undergone surgical resection [18]. In their study, histopathological findings and RFS were not statistically different. In our study, the histopathological findings between solitary AD and SQ showed similar outcomes. However, the SQ group was associated with shorter overall survival (OS) than the AD group (*p* < 0.01). We suspect that, in the SQ group, patient characteristics, such as elderly age, higher incidence in the male sex, and heavy smoking history were associated with a higher rate of death from other disease than AD group. Sakurai evaluated 70 peripheral SQ with 1–3 cm in diameter [19]. The 5-year OS and RFS rates were 73.4% and 85.9%, respectively; 53% died due to cancer recurrence, while 47% succumbed to other diseases. Considering the high prevalence of non-cancerous deaths in patients with high-risk characteristics like the SQ group, surgeons should take more caution while formulating a surgical plan, taking into account the patent’s physical characteristics and risk factors.

The SUVmax is the most frequently evaluated prognostic factor of positron emission tomography-CT (PET-CT). Ito et al. reported that SUVmax was significantly correlated with tumor recurrence in the cohort of solitary AD and SQ (*p* = 0.004) [18]. Ichikawa et al. evaluated 50 patients with pure solid AD in clinical stage IA [20]. They found that SUVmax was an independently significant prognostic factor. The five-year OS and RFS rates of the SUV max ≥5.4 and <5.4 groups were 68.0% versus 100%, and 54.3% versus 90.8% (*p* = 0.002, and <0.001). Bayarri-Lara et al. also suggested that SUVmax was an independent predictor for the presence of postoperative circulating tumor cells, which were significantly correlated with a shorter RFS (*p* = 0.005) [21]. In our cohort, pathological type was not associated with cancer recurrence. On preoperative parameters, whole tumor size on lung window setting (WTS), MD, or TDR were not prognostic factors (*p* = 0.127, 0.066, and 0.082), but higher value of SUVmax was most useful parameter to predict cancer recurrence (*p* = 0.016). In this study, the optimal cut-off value for predicting cancer recurrence was 4.6 (area under the curve = 0.674; sensitivity = 94.7%; specificity = 69.4%; 95% confidence interval = 0.564–0.780; *p* = 0.016). SUVmax <4.6 was associated with longer RFS; however, the value was not associated with CSS (log-rank test: *p* = 0.201). Hyun et al. evaluated some radiological tools using SUV as continuous variables after adjusting for age, sex, histology, tumor stage, and type of surgery [22]. They reported that SUVmax showed statistical tendency to RFS (*p* = 0.056), but not a prognostic factor for OS (*p* = 0.525). Additionally, metabolic tumor volume (MTV), and total legion glycolysis (TLG) were associated with an increased risk of recurrence (*p* = 0.001; MTV, *p* < 0.001; TLG) and death (*p* = 0.009; MTV, *p* = 0.007; TLG). However, these parameters are still less versatile evaluation methods and required system construction and future research. In summary, SUVmax was useful for predict RFS, but was controversial for OS. In pure solid AD and SQ form larger than 10 mm to 30 mm, clinicians should consider aggressive surveillance or indication of aggressive adjuvant therapy for tumors with SUVmax ≥4.6, apart from upstaging. In the future, further screening studies for personalized treatment will need to be established, such as the evaluation of perioperative circulating tumor cells in patients with high SUVmax values.

Subsequently, we analyzed risk factors for pathological LNM. Regarding tumor markers, a high value of CYFRA was statistically associated with LNM. However, the difference was slight and not clinically significant. The short-axis diameter of an LN <10 mm on axial CT is generally considered as clinically negative LNM. The false positive and false negative rates were roughly 40% and 20%, respectively [23]. In recent reports, SUVmax of primary lesion was useful as the independent predictor of LNM in lung cancer. Park et al. suggested that SUVmax >7.3 in primary tumors offered an independent predictor of occult nodal metastasis in patients with clinical stage IA NSCLC [24]. Kaseda et al. suggested that the optimal cut-off for SUVmax of the primary tumor for LNM determined from the ROC curve was 3.0 in the clinical stage I NSCLC [25]. Nambu et al. reported that there was no LNM in cases with a tumor SUVmax <2.5 [26]. In this study, SUVmax ≥4.6, which evaluated as predictive value for cancer recurrence, showing a tendency of LNM. Additionally, the quantitative continuous variables using any radiological tools were not associated with LNM. However, MD ≥8 mm with SUVmax ≥2.4 could be a risk factor for LNM. PET-CT is a functional imaging modality and as PET-CT methodologies vary at different institutions; clinicians should not overlook the differences in SUVmax levels [27]. Therefore, our data showed that MD <8 mm using HRCT was useful as the other predictive parameter of non-LNM. Finally, we suggested that both SUVmax and MD could be useful for surgical planning.

This study has several limitations. First, this investigation was a retrospective observation in a single facility, and the generalizability of the findings is limited. Second, pathological findings and mutation status are important to predict prognosis. However, we did not compare radiological tools and pathological findings according to the aim of our study. Third, lobectomy, and sublobar resection were included in the study as surgical procedures. However, it was not identified how this election occurred. Fourth, analysis of pathological inflammation and c-reactive protein level at the time of PET CT may affect the outcomes. However, these data were lacking in this study.

## 5. Conclusions

Radiologically pure solid AD and SQ were equivalent for the RFS and CSS. However, OS in the SQ group was poorer due to more non-cancerous deaths than AD group. SUVmax ≥4.6 was useful to predict recurrence, regardless of parameters using HRCT. Tumor diameter on mediastinal setting ≥8 mm with SUVmax ≥2.4 could be a risk factor for LNM.

## Figures and Tables

**Table 1:** Comparison between adenocarcinoma (AD) and squamous cell carcinoma (SQ).

Variables	Overall (*n* = 130)	AD Group (*n* = 96)	SQ Group (*n* = 34)	*p*-Value
	*N* (%), Median or Average ± SD, Range	*N* (%), Median or Average ± SD, Range	*N* (%), Median or Average ± SD, Range	
Age (year old)	69 ± 8.6 (43–86)	68 ± 8.9 (43–84)	75 ± 6.1 (57–86)	<0.01 ^a^
Sex (male)	69 (53.1%)	39 (40.6%)	30 (88.2%)	<0.01 ^b^
Smoking index	625 ± 736 (0–3600)	7 ± 663 (0–2950)	1030 ± 700 (0–3600)	<0.01 ^a^
Spirometry test				
%VC	98 ± 13.8 (67.8–149)	99.6 ± 13.7 (68.9–149)	95 ± 13.6 (67.8–120.7)	0.058 ^a^
FEV1.0%	76.5 ± 8.5 (46–98)	77.3 ± 9.1 (46–98)	74 ± 6.5 (59.5–88.8)	0.128 ^a^
Findings on CT				
whole tumor size (mm)	19.9 ± 5.2 (11–30)	20 ± 5.1 (11–30)	18.9 ± 5.5 (11–30)	0.484 ^a^
mediastinal diameter (mm)	15.7 ± 5.9 (0–29)	15.4 ± 6 (0–27)	16.4 ± 5.4 (7–29)	0.398 ^d^
TDR (%)	15.8 ± 19 (0–100)	17.2 ± 20.6 (0–100)	14.3 ± 12 (0–47.3)	0.048 ^a^
SUVmax	7.7 ± 7.2 (1.5–33.9)	6.4 ± 6.2 (1.5–33.9)	14 ± 7.6 (3.6–30.7)	<0.01 ^a^
Lobectomy/sublobar resection	95/35 (73.1/26.9%)	70/26 (72.9/26.1%)	25/9 (73.5/26.5%)	0.945 ^b^
Mediastinal lymph node dissection	90 (69.2%)	68 (70.8%)	22 (64.7%)	0.506 ^b^
Pathological invasive size (mm)	17 ± 7.2 (1–44)	16 ± 6.9 (1–40)	18 ± 8 (4–44)	0.349 ^a^
Lymphovascular invasion	100 (76.9%)	74 (77.1%)	26 (76.5%)	0.942 ^b^
Pleural invasion (pl2)	10 (7.7%)	9 (9.4%)	1 (2.9%)	0.208 ^c^
Histological low grade	32 (24.6%)	26 (27.1%)	6 (17.6%)	0.272 ^b^
Pathological lymph node metastasis	19 (14.6%)	13 (13.5%)	6 (17.6%)	0.571 ^b^
Adjuvant chemotherapy	10 (7.7%)	7 (7.3%)	3 (8.8%)	0.528 ^c^
Cancer recurrence	19 (14.6%)	15 (15.8%)	4 (11.8%)	0.408 ^c^

%VC, percent of vital capacity; FEV1.0%, forced expiratory volume in 1 s as a percent of forced vital capacity; TDR, tumor disappearance ratio; SUV, standardized uptake value; ^a^, Mann–Whitney test; ^b^, Chi-square test; ^c^, Fisher exact test; ^d^, Student *t*-test.

**Table 2:** Comparison with recurrence group and non-recurrence groups.

Variables	Recurrence Group (*N* = 19)	Non-Recurrence Group (*N* = 111)	*p*-Value
	*N* (%), Median ± SD, Range	*N* (%), Median ± SD, Range	
Age (year old)	67 ± 7.8 (53–81)	69 ± 8.8 (43–86)	0.649 ^a^
Sex (male)	11 (57.9%)	66 (59.5%)	0.898 ^b^
Smoking index	720 ± 764 (0–2580)	620 ± 735 (0–3600)	0.730 ^a^
Findings on CT			
whole tumor size (mm)	23.6 ± 5.6 (11–29)	19 ± 5.1 (11–30)	0.127 ^a^
mediastinal diameter (mm)	18 ± 5.7 (7–27)	16 ± 5.8 (0–29)	0.066 ^c^
tumor disappearance ratio (%)	9.9 ± 15.3 (0–61.5)	16.7 ± 19.5 (0–100)	0.082 ^a^
SUVmax	11.5 ± 6.3 (4.7–28.3)	6.9 ± 7.3 (1.5–33.9)	0.016 ^a^
Spirometry test			
%VC	100 ± 16.3 (67.8–127.5)	98 ± 13.5 (68.9–149)	0.642 ^a^
FEV1.0%	77.4 ± 8.1 (54.8–91.5)	76.4 ± 8.7 (46–98)	0.767 ^a^
CEA (ng/mL)	3.2 ± 9.3 (0.8–35.3)	3.3 ± 3.4 (0.5–20.1)	0.395 ^a^
CYFRA (ng/mL)	1.4 ± 0.7 (0.6–2.8)	1.3 ± 0.7 (0.4–3.9)	0.806 ^a^
Lobectomy/sublobar resection	15/4 (78.9/21.1%)	80/31 (72.1/27.9%)	0.532 ^b^
Mediastinal lymph node dissection	11 (57.9%)	79 (71.2%)	0.247 ^b^
Squamous cell carcinoma	4 (21.1%)	30 (27%)	0.408 ^d^
Pathological whole size (mm)	21 ± 7.8 (4–40)	18 ± 6.2 (8–44)	0.034 ^a^
Pleural invasion (pl2)	2 (10.5%)	8 (7.2%)	0.446 ^d^
Histological low grade	8 (42.1%)	24 (21.6%)	0.056 ^d^
Lymph node metastasis	7 (36.8%)	12 (10.8%)	0.007 ^d^
Adjuvant chemotherapy	3 (15.8%)	7 (6.3%)	0.140 ^d^

%VC, percent of vital capacity; FEV1.0%, forced expiratory volume in 1 s as a percent of forced vital capacity; ^a^, Mann–Whitney test; ^b^, Chi-square test; ^c^, Student *t*-test; ^d^, Fisher exact test.

**Table 3:** Analysis of lymph node metastasis (LNM).

Variables	LNM Group (*N* = 15)	Non-LNM Group (*N* = 68)	*p*-Value
	*N* (%), Median or Average ± SD, Range	*N* (%), Median or Average ± SD, Range	
Age (year old)	66 ± 8.5 (47–79)	68 ± 7.7 (43–80)	0.424 ^a^
Sex (male)	6 (40%)	38 (55.9%)	0.265 ^b^
Smoking index	0 ± 586 (0–1920)	645 ± 684 (0–3000)	0.080 ^a^
Findings on CT			
whole tumor size (mm)	21 ± 5.5 (11–28)	20 ± 4.8 (12–30)	0.526 ^a^
mediastinal diameter (mm)	16.9 ± 6 (8–25)	16 ± 5.7 (0–27)	0.567 ^c^
tumor disappearance ratio (%)	14.3 ± 15 (5.3–56.8)	15.4 ± 20.4 (0–100)	0.939 ^a^
SUVmax	8.9 ± 6.9 (2.4–30.2)	7.7 ± 7 (1.5–33.9)	0.558 ^a^
SUVmax ≥4.6	14 (93.3%)	49 (72.1%)	0.072 ^d^
CEA (ng/mL)	3.3 ± 4.7 (0.8–20.1)	3.3 ± 3.9 (0.6–25.9)	0.892 ^a^
CYFRA (ng/mL)	1.2 ± 0.6 (0.4–3)	0.8 ± 0.5 (0.5–2.3)	0.041 ^a^
Pathological whole size (mm)	21 ± 9.2 (8–44)	19 ± 5.6 (8–40)	0.726 ^a^
Pathological invasive size (mm)	20 ± 9.4 (8–44)	17.5 ± 6.4 (1–40)	0.529 ^a^
Squamous cell carcinoma	3 (20%)	17 (25%)	0.485 ^d^
Total number of excised lymph nodes	15 ± 5.8 (7–26)	13 ± 6.5 (5–32)	0.803
Lymphvascular invasion	15 (100%)	50 (73.5%)	0.017 ^d^
Pleural invasion (pl2)	2 (13.3%)	7 (10.3%)	0.513 ^d^
Histological low grade	5 (33.3%)	13 (19.1%)	0.191 ^d^

^a^, Mann–Whitney test; ^b^, Chi-square test; ^c^, Student *t*-test; ^d^, Fisher exact test.

## Data Availability

The data presented in this study are available in Result part.
